# Neolignans isolated from industrial hemp (*Cannabis sativa* L.) roots have cytotoxic effects on cancer cells

**DOI:** 10.1186/s42238-025-00316-5

**Published:** 2025-08-16

**Authors:** Korey J. Brownstein, Grace E. Nieukirk, Jackson Edwards, Maria Thomas, Thu Hien Nguyen, Pedro A. de Alarcón, Karl E. Vermillion, Manu Gnanamony

**Affiliations:** 1 USDA, ARS, NCAUR, Functional Foods Research Unit, Peoria, IL 61604 USA; 2 U.S. Department of Energy, Oak Ridge Institute for Science and Education (ORISE) appointment, Oak Ridge, USA; 3 Department of Pediatrics, University of Illinois College of Medicine Peoria, One Illini Drive, Peoria, IL 61605 USA

**Keywords:** *Cannabis sativa*, Industrial hemp, Neolignan, Root, Pediatric cancer

## Abstract

**Background:**

The 2018 Farm Bill states that cultivars of *Cannabis sativa* L. (industrial hemp) are legal for industrial use if total tetrahydrocannabinol (THC) concentrations are less than 0.30%. Due to this legislation, hemp cultivars with low total THC have found a wide range of uses, from animal feed to paper production. Although cannabinoids are the most widely studied compounds in hemp, hemp produces numerous other compound classes as well, and these phytochemicals may have uses in the functional food and pharmaceutical industry.

**Methods:**

Initial liquid chromatography profiling of hemp root samples revealed a group of uncharacterized peaks, and these peaks were tentatively identified as neolignans by Oribitrap ID-X high resolution mass spectrometer. To further elucidate the structure of these neolignans, we used techniques in liquid-liquid extraction, as well as flash chromatography to isolate them in preparation for NMR analysis. We then tested their inhibitory concentration 50 (IC50) in a variety of cancer cell lines.

**Results and discussion:**

Four neolignans were isolated from hemp roots and each differed in their molecular weight by 30 daltons. Two of the compounds were identified as dadahols A and B. We tested fractions of various purities containing neolignans against neuroblastoma cell lines CHLA15 and LAN5, hepatoblastoma cell line Hep3B, and Hodgkin’s lymphoma cell line L428. We found that semi-pure fractions containing dadahol A and/or dadahol B had the highest cytotoxic activity. We then tested pure dadahol A and dadahol B, and this revealed dadahol A exhibited the lowest IC50 values in all the cell lines.

**Supplementary Information:**

The online version contains supplementary material available at 10.1186/s42238-025-00316-5.

## Introduction

Industrial hemp (*Cannabis sativa* L.), which has less than 0.30% total tetrahydrocannabinol (THC) is being studied for a variety of applications besides being a source for cannabidiol (CBD). Applications include animal feed, paper production, and use in building materials, among others. However, most researchers investigate the aerial parts of hemp to characterize its phytochemical composition and/or industrial applications. For the small number of researchers characterizing the chemical composition of hemp roots, a wide range of compound classes have been shown to be present, such as cannabinoids, flavonoids, and triterpenes (Huang et al. 2023; Jin et al. 2020; Stout et al. 2012). Several lignans have also been identified in hemp roots by Huang et al. (2023). One lignan, dadahol A, described as a neolignan and first isolated from *Artocarpus dadah* Miq. (Su et al. 2002) was found in hemp roots as well (Huang et al. 2023). Su et al. (2002) and Huang et al. (2023) reported that dadahol A was inactive in inflammation assays. Here, we describe several new neolignans isolated from hemp roots for the first time exhibiting antiproliferation activities on cancer cells.

## Materials and methods

### Extraction of neolignans from hemp roots

An amount of 300 mL of LC-MS grade methanol was added to 40 g of hemp roots in a glass beaker. Afterwards, the beaker was covered with foil and sonicated at 40 kHz for 30 min in a Bransonic sonication bath (Emerson Electric Co., St. Louis, MO, USA). The extract was decanted into a second beaker. Another 300 mL of methanol was added to the beaker with the root materials, sonicated for 30 min, and then decanted into the second beaker; this step was repeated twice more. The combined extracts were dried down to about 100 mL in a fume hood for 3 days at room temperature. After 3 days, 160 mL of methanol: 18 MΩ water [1:1] was added to the beaker. This solution was swirled, placed in a sonication bath for 30 min, and then filtered through a 0.45-µm nylon filter into a new beaker. After allowing the solution to dry down to about 100 mL in a fume hood for 3 days at room temperature, another 160 mL of methanol: 18 MΩ water [1:1] was added, and the solution was again swirled and sonicated for 30 min. The solution was filtered one final time through a 0.45-µm nylon filter into a separatory funnel. Ethyl acetate (100 mL) was added to the separatory funnel and the funnel was inverted five times. Once there was a clear separation of two phases, the ethyl acetate (top) phase was transferred to a beaker using a glass Pasteur pipette. Another 100 mL of ethyl acetate was then added to the funnel. After inverting five times, this ethyl acetate phase was combined with the other ethyl acetate phase.

### Purification of hemp neolignans by flash chromatography

The ethyl acetate phase (150 mL) was dried in the hood for 1 day. Afterwards, the dried extract was resuspended with 100 mL of methanol, and water was added dropwise until the extract started turning cloudy. The first step in purification used a Büchi Sepacore flash chromatography system (New Castle, DE, USA) with dual C-605 pump modules, C-620 control unit, C-635 UV photometer and C-660 fraction collector all controlled by the SepacoreControl chromatography software v1.4.3000.18063 standard edition. The column was a 90 g SiliaSep BU C18 reverse phase flash column (230–400 mesh, 40–63 μm, Silicycle, QC, Canada). The column was conditioned with 70% water containing 0.10% acetic acid (A) and 30% methanol (B) at a flow rate of 50 mL/min for 5.00 min. After injection of the samples (20 mL at a time), the following gradient setup was implemented: 70% A:30% B from 0.00 to 5.00 min followed by a gradual change from 70% A:30% B to 0% A:100% B between 5.00 and 25.00 min. The effluent was monitored at 280 nm, and, after a 20.00 min delay, the fractions were automatically collected by time (30 s). Column cleaning was then performed with 100% B for 10 min. The procedure was repeated until all the extract was loaded/injected. The fractions with the single, largest UV absorbing peak were pooled and evaporated in the fume hood to remove the acetic acid and methanol, which allowed for the formation of solid white crystals. The remaining water in the pooled fraction was removed by lyophilizing it for 3 days.

The lyophilized sample (5 mg) was then dissolved in 50 mL of chloroform: methanol [25: 2] and subjected to a second round of purification using a Rheodyne Peek load/inject valve (IDEX Corporation, Northbrook, IL, USA) connected to an Agela Cheetah MP200/MP100 flash chromatography system (Torrance, CA, USA). The Agela system was controlled by Agela Technologies software v3.1.2. The column was an 80 g FlashPure Büchi Silica 40-µm irregular flash column (New Castle, DE, USA). The column was conditioned with 100% chloroform: methanol [25: 2] at a flow rate of 20 mL/min for 5.00 min. After injection of the samples (10 mL at a time), the following isocratic setup described in Slanina and Glatz (2004) was implemented: 100% chloroform: methanol [25: 2] from 0.00 to 20.00 min. The effluent was monitored at 280 nm, and, after a 5.00 min delay, the fractions were automatically collected by volume (2 mL) until 14 min. Under our conditions, the fractions containing the major UV absorbing peaks spanned between fractions 20 and 60. Column cleaning was then performed with 100% chloroform: methanol [25: 2] for 10 min. The procedure was repeated until all the sample was loaded/injected. The fractions containing UV absorbing peaks were dried in the fume hood for 1 day revealing white crystals. Afterwards, all the fractions were resuspended with 200 µL of methanol and analyzed by liquid chromatography-mass spectrometer.

### Liquid chromatography-mass spectrometer analysis of hemp neolignans

Liquid chromatography (LC) was performed on a Thermo Scientific Vanquish ultra-high-performance liquid chromatography (UHPLC) system (Thermo Fisher Scientific Inc., Waltham, MA, USA) with photodiode array (PDA) detection ranging between 210 and 400 nm. Ten microliters of sample were injected into a 25.0 µL sample loop, and the flow rate through an Inertsil ODS-3, 3 μm, 3.0 × 150 mm column (GL Sciences, Torrance, CA, USA) was 0.60 mL/min. The linear gradient elution using 0.10% formic acid/water (A) and 0.10% formic acid/methanol: acetonitrile [1:1] (B) was applied as follows: initial conditions of 65% A:35% B from 0.00 to 2.00 min, changed gradually from 65% A:35% B to 25% A:75% B between 2.00 and 16.00 min, maintained at 25% A:75% B until 18.00 min, increased to 0% A:100% B from 18.00 to 19.00 min, held at 0% A:100% B for 10.00 min, returned to the initial conditions of 65% A:35% B in 1.00 (30.00) min, and then, before the next injection, maintained at 65% A:35% B until 35.00 min. The analysis time was 35.00 min. The autosampler chamber, column chamber, pre-heater, and post column cooler temperatures were set at 8, 50, 50, and 40 °C, respectively.

A Thermo Scientific Orbitrap ID-X Tribrid mass spectrometer (MS) was operated using heated-electrospray ionization (H-ESI) in the polarity switching mode, i.e., both negative and positive modes in a single run. The Orbitrap resolution for mass spectral features was 120,000, and the scan range was from 150 to 2,000 *m/z* with the RF lens set to 60%. MS^n^ data, using a resolution of 60,000, were collected for mass spectral features with an intensity threshold greater than 2.0e4. For dynamic exclusion, mass spectral features limited to a mass tolerance of 5 ppm were excluded after 1 time for 60 s. With an isolation window of 2 *m/z*, higher-energy collisional dissociation (HCD) collision energies of 15%, 30% and 45% were used in stepped mode. These parameters were executed in 0.6 s. The spray voltages were 3,450 V for positive ions and 2,250 V for negative ions. The sheath, aux, and sweep gases were set to 70, 20, and 10 arbitrary units, respectively. The ion transfer tube temperature was 350 °C, while the vaporization temperature was 400 °C.

Water (18 MΩ) was obtained from a Barnstead GenPure Pro water purification system (Thermo Fisher Scientific Inc., Waltham, MA, USA). Ethyl acetate, chloroform, glacial acetic acid, Optima acetonitrile and Optima methanol were purchased from Fisher Chemical (Waltham, MA, USA), while Fluka formic acid was purchased from Honeywell (Charlotte, NC, USA). All solvents used for analysis were of analytical chemistry grade or higher.

### Processing of MS^n^ data

MS^n^ spectrum of individual mass spectral features was converted to an.MGF file using MSConvert (Chambers et al. 2012). Afterwards, the.MGF files were uploaded to SIRIUS v5.8.6 (Dührkop et al. 2019), and the predicted formula and structure of each mass spectral feature was determined in “Compute All.” In molecular formula identification, the parameters were set with instrument: Orbitrap; MS2 mass accuracy (ppm): 5; possible ionizations: [M-H]^−^; element H from 0 to infinity; element C from 0 to infinity; element N from 0 to infinity; element O from 0 to infinity; element P from 0 to infinity; and element S from 0 to infinity. In fingerprint prediction, the fallback adducts were [M-H]^−^ and [M + CH202-H]^−^. In structure database search, all databases were selected. Under our conditions, dadahol A (697.2289 [M-H]^−^ *m/z*) eluted at around 14.33 min. A commercially available reference standard of dadahol A (TargetMol, Wellesley Hills, MA, USA) was used to confirm the identity of dadahol A. Each fraction (F) between 20 and 60 was analyzed by LC-MS followed by processing in SIRIUS to predict the chemical composition of the fractions.

### Nuclear magnetic resonance of hemp neolignans

All NMR spectra were collected with a JEOL ECZ600R spectrometer (600.17 MHz, Peabody, MA, USA) using a 5 mm cryoprobe probe. All samples were dissolved in MeOD-d4 and all spectra were acquired at 25 °C. Chemical shifts are reported as ppm from tetramethylsilane calculated from the lock signal. Spectra were processed with the JEOL Delta software v6.2.

### Cell lines and media

We used four cancer cell lines to study the effect of these compounds on the viability of cancer cells—CHLA15, LAN5, Hep3B and L428. CHLA15 and LAN5 cell lines were acquired from the Children’s Oncology Group (COG) childhood cancer repository and were cultured in Roswell Park Memorial Institute (RPMI) 1640 medium. Hep3B purchased from the ATCC, and L428 (gifted by Dr. Ralf Kueppers, University of Essen, Germany) were cultured in Dulbecco’s Modified Eagle’s Medium (DMEM). All these cancer cell lines are well-established models with documented and consistent sensitivity to standard cytotoxic agents (D’Souza et al. 2022; Gnanamony et al. 2025; Meng et al. 2011). The media were supplemented with 10% fetal bovine serum (FBS) and 1% penicillin-streptomycin. Cells were grown at 37 °C in a CO_2_ incubator. Mycoplasma testing was periodically done using Mycoplasma PCR Detection and Elimination Kit (ABM, Richmond, BC, Canada).

Semi-pure extracts, 757/727, 757/727/697, 727/697, and 697/667, which are labelled hereafter as M1, M2, M3, and M4, respectively, were dissolved in dimethyl sulfoxide (DMSO) at a concentration of 10 mg/mL. F43/44 (containing dadahol B) and dadahol A reference standard (697.2289 [M-H]^−^ *m/z*) were dissolved in DMSO at a concentration of 10 mM. The compounds were stored in multiple aliquots at -20 °C protected from light. For experiments, the compounds were diluted in cell culture media as required.

### Cell viability

Resazurin Cell Viability Assay Kit (Biotium, Fremont, CA, USA) was used to assess the effect of the compounds on cancer cell viability. Cells were seeded onto 96 well tissue culture plates at appropriate densities—CHLA15 (1.75 × 10^4^), LAN5 (2 × 10^4^), Hep3B (0.5 × 10^4^) and L428 (1.25 × 10^4^) and allowed to adhere and grow for 24 h. Following treatment with the appropriate compound, the plates were incubated for 72 h in a CO_2_ incubator. Subsequently, 10 µL of resazurin dye was added to all the wells and incubated for an additional four hours in a CO_2_ incubator. Absorbance was measured at 570 nm with a correction at 600 nm using an xMark Microplate Spectrophotometer (Bio-Rad, Hercules, CA, USA). Corrected absorbance values less than zero which indicates complete absence of viable cells were set to zero to facilitate ease of data interpretation. Percent viability was calculated in Microsoft Excel. Statistical significance was determined for compounds that caused at least 50% inhibition of cell viability. For inhibitory concentration 50 (IC50) determination, cells were treated with 5-fold dilutions of compounds. After 72 h, cell viability was determined as described above.

### Statistical analysis

Statistical analyses for all the biological assays were conducted, and graphs were created using GraphPad Prism v10 (Dotmatics, Boston, MA, USA). Viability assays and IC50 analysis were performed in at least three replicates. For multigroup analyses, one-way ANOVA with Dunnett post hoc correction was used. A *p*-value ≤ 0.05 was considered statistically significant.

## Results and discussion

**Fig. 1:**
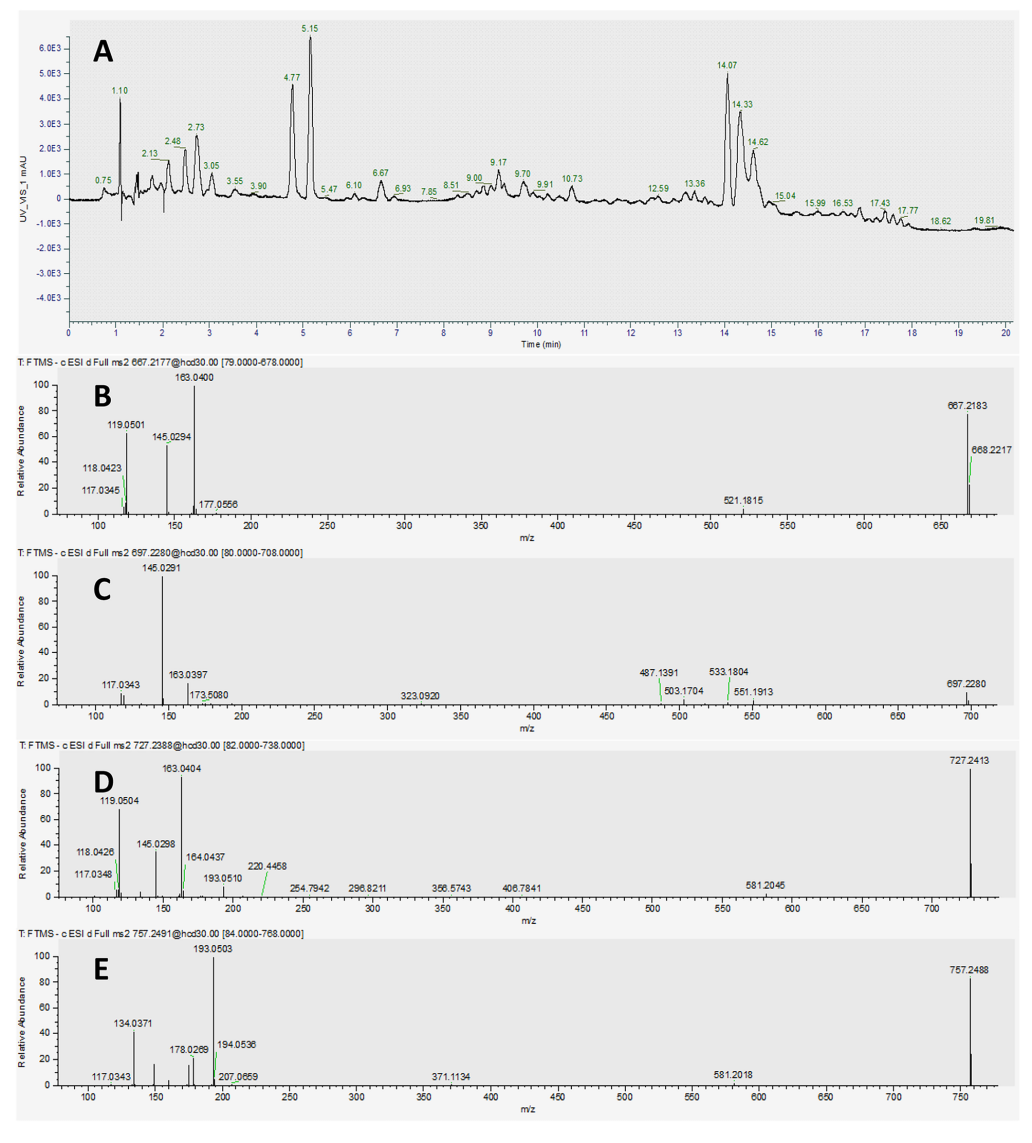
Liquid chromatography-mass spectrometer (LC-MS) analysis of the ethyl acetate phase monitored at a wavelength of 340 nm. (**A**) Analysis of the four peaks at 14.07, 14.33, 14.62, and 15.04 min revealed the following mass spectral features in eluting order: 667.2180 *m/z*, 697.2289 *m/z*, 727.2388 *m/z*, and 757.2490 *m/z*. The MS^n^ spectra of 667.2180 *m/z* (**B**), 697.2289 *m/z* (**C**), 727.2388 *m/z* (**D**), and 757.2490 *m/z* (**E**) were also collected by the MS

LC-MS analysis revealed that F28 (< 0.5 mg), F31 (< 0.5 mg), F35/36 (< 0.5 mg), and F43/44 (0.5 mg) contained the following mass spectral features: 757.2490 [M-H]^−^ *m/z*, 727.2388 [M-H]^−^ *m/z*, 697.2289 [M-H]^−^ *m/z*, and 667.2180 [M-H]^−^ *m/z*, respectively. The analysis also showed that there were fractions with mixtures of 757/727 (M1), 757/727/697 (M2), 727/697 (M3), and 697/667 (M4). The mass spectral features 757.2490 *m/z*, 727.2388 *m/z*, 697.2289 *m/z*, and 667.2180 *m/z* eluted at around 15.04, 14.62, 14.33, and 14.07 min, respectively (Fig. 1). Importing the MS^n^ of 697.2289 *m/z* into SIRIUS tentatively identified this mass spectral feature as dadahol A. We then procured a reference standard of dadahol A (TargetMol, Wellesley Hills, MA, USA), which enabled us to confirm that F35/36 contained dadahol A.

To further characterize the composition of F28, F31, and F43/44, we performed NMR analysis, and this revealed varying degrees of methoxy (-CH_3_O) addition and subtractions from dadahol A. That is, F28 had two methoxy additions, while F31 had one methoxy addition. The mass spectral feature in F43/44 had one less methoxy than dadahol A. These data explain the 30 dalton differences we observed in the mass spectrometer. After interpretation of various NMR spectra, we could not confidently determine the placement of the methoxy groups for 757.2490 *m/z* and 727.2388 *m/z*. This was probably due to having a limited amount of sample (< 0.5 mg) present in F28 and F31. However, we were able to determine that F43/44 contained another dadahol previously reported in the literature as dadahol B (Su et al. 2002). This is the first report of dadahol B being present in hemp.

Comparison of our NMR spectra data to the data published in Su et al. (2002) revealed several discrepancies. First, carbons C-2 and C-2’ were listed in two different places with different shifts, once as 119.3 and 119.0 ppm and again later as 111.7, 111.6, and 111.5 ppm. Our data supported the latter set. Second, in Su et al. (2002), the carbon shifts for the two isomers at C-8 were listed as 80.4 and 83.6 ppm, but all other carbon peaks are listed in descending order. Our data supports a transposition of the first number, 84.0 and 83.6. Lastly, the shifts for the methoxy protons are listed as two different shifts, one for each of the two different sets of protons. Su et al. (2002) reported a 50/50 mix of isomers. Thus, it is possible that both methoxy groups have the same shift and the two isomers are different, or the two methoxy groups are different, and the isomers do not influence their positions. Since our methoxy protons are in a 3:1 ratio, it is more likely that the two methoxy peaks have the same shift in each isomer, but the two peaks are due to the erythro/threo pair. Overall, our data agree well with that of the Su et al. (2002) data (Supplementary Table 1). Our data also show a doubling of the C-7 and C-8 peaks in a 1:1 ratio, which matches that of Su et al. (2002). NMR analysis had also determined that F43/44 was 80% dadahol B and 20% dadahol A.

**Fig. 2:**
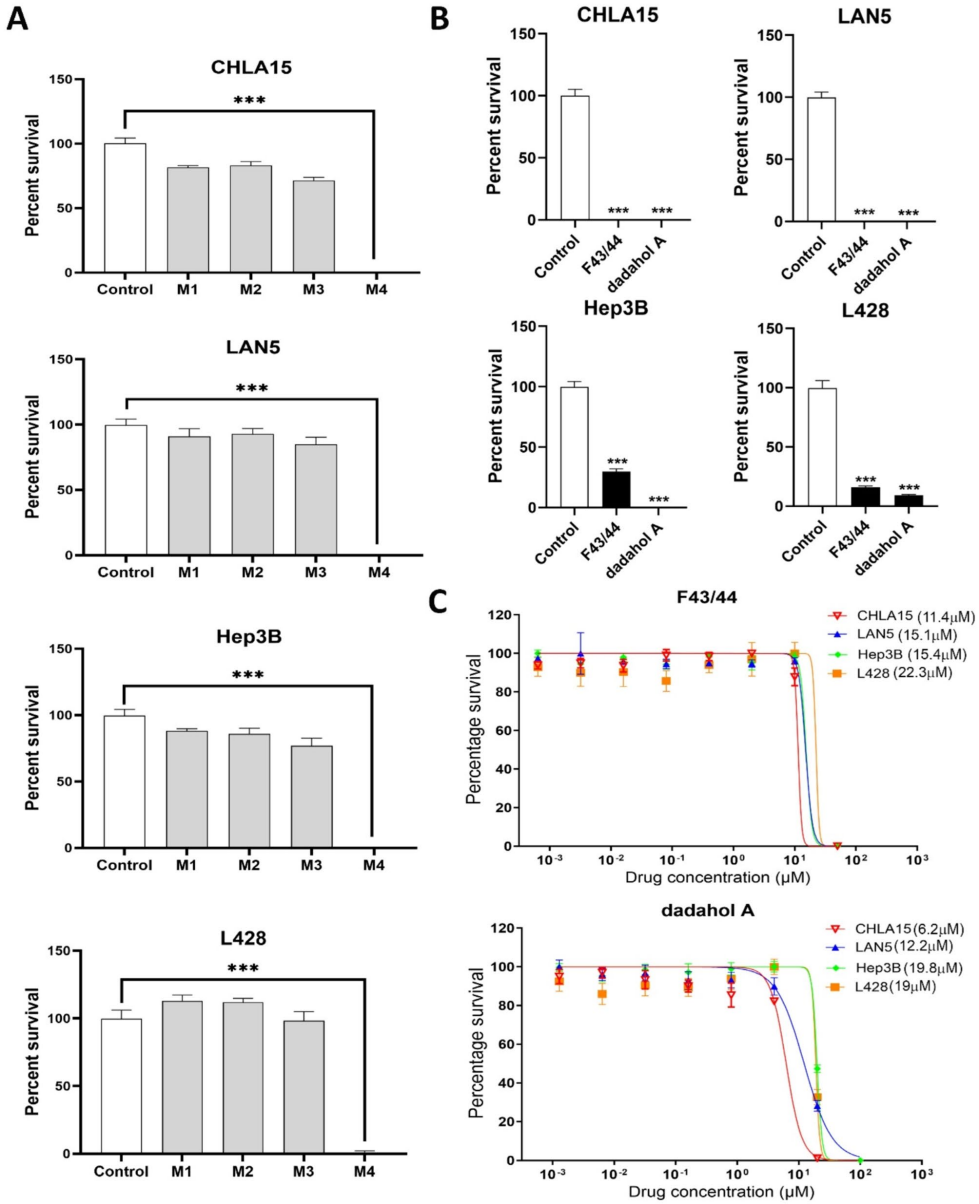
Cytotoxicity of hemp root fractions and commercially available dadahol A on different cancer cell lines. (**A**) Graph showing cell viability after treatment with hemp fractions (100 µg/mL) in CHLA15, LAN5, Hep3B, and L428 cell lines. (**B**) Graph showing viability after treatment with F43/44 (25 µM) and dadahol A (25 µM) in CHLA15, LAN5, Hep3B, and L428 cell lines. (**C**) Survival graph showing inhibitory concentration 50 (IC50) of F43/44 and dadahol A in CHLA15, LAN5, Hep3B, and L428 cell lines. *** *p* < 0.001

To explore the antiproliferation activities of the hemp root extracts, we initially screened the semi-pure extracts (i.e., M1, M2, M3, and M4) for their effect on cancer cell viability at a fixed concentration of 100 µg/mL across four different cancer cell lines. The four cell lines were selected based on their relevance to pediatric cancers and their well-established use in preclinical studies modeling childhood malignancies. These included two cell lines derived from high-risk neuroblastoma, a common extracranial childhood tumor (CHLA15 and LAN5), and one each from pediatric liver cancer (Hep3B) and Hodgkin’s lymphoma (L428). Among the four extracts screened, M4 caused significant inhibition of cell survival (*p*-value < 0.001) in all four cancer cell lines in comparison to the control (Fig. 2A).

Since M4 contained two different compounds, we aimed to determine whether the observed effect was due to one specific compound or both. To achieve this, F43/44 (containing dadahol B) and commercially available dadahol A were then tested individually to assess their effects on the viability of cancer cells at 25 µM. Interestingly, both compounds demonstrated a significant reduction in cell viability compared to the control in all four cancer cell lines indicating that both these compounds have the potential to be used as antiproliferation agents (Fig. 2B).

To quantify their growth inhibitory potency, we determined inhibitory concentration 50 (IC50) values for both F43/F44 (dadahol B) and dadahol A (Fig. 2C). Both compounds exhibited the lowest IC50 values against neuroblastoma cell lines CHLA15 (F43/44 at 11.4 µM and dadahol A at 6.2 µM) and LAN5 (F43/44 at 15.1 µM and dadahol A at 12.2 µM), followed by hepatoblastoma cell line Hep3B (F43/44 at 15.4 µM and dadahol A at 19.8 µM) and the Hodgkin’s lymphoma cell line L428 (F43/44 at 22.3 µM and dadahol A at 19 µM). This suggests that the compounds isolated from hemp root vary in their efficacy against different cancers. Su et al. (2002) and Huang et al. (2023) have described dadahols as inactive in their inflammation assays. We report, for the first time, that dadahols, using the methodologies described herein, have antiproliferative effects. While our findings demonstrate the cytotoxic effects of hemp-derived compounds on multiple pediatric cancer cell lines, the underlying mechanisms driving these effects remain to be elucidated. Future studies will focus on detailed mechanistic investigations including cell cycle analysis, apoptosis and necrosis assays, and pathway-level interrogations to uncover the molecular basis of this cytotoxicity.

Dadahols A and B are two neolignans that were previously characterized by Su et al. (2002) in the twigs and branches of *A. dadah* (Moraceae family). Although *Cannabis* is in the Cannabaceae family, it is a member of the order Rosales, which includes *A. dadah*. In fact, a publication by Huang et al. (2023) revealed that dadahol A is present in hemp roots. Though industrial hemp is more well-known for its cannabinoid content, it contains hundreds of other phytochemicals that may have uses in the functional food and pharmaceutical industry. Furthermore, utilizing each part of the hemp plant, including the roots, will reduce agricultural waste and provide additional value to growers.

## Supplementary Information

Below is the link to the electronic supplementary material.

Supplementary Material 1

## Data Availability

No datasets were generated or analysed during the current study.
